# Downregulation of mGluR1-mediated signaling underlying autistic-like core symptoms in *Shank1* P1812L-knock-in mice

**DOI:** 10.1038/s41398-023-02626-9

**Published:** 2023-10-25

**Authors:** Yue Qin, Xiao-Yong Zhang, Yanyan Liu, Zehan Ma, Shuo Tao, Ying Li, Rui Peng, Fei Wang, Jiucun Wang, Jianfeng Feng, Zilong Qiu, Li Jin, Hongyan Wang, Xiaohong Gong

**Affiliations:** 1 State Key Laboratory of Genetic Engineering, School of Life Sciences, Obstetrics and Gynecology Hospital, Fudan University, Shanghai, China; 2 Institute of Science and Technology for Brain-Inspired Intelligence, Fudan University, Shanghai, China; 3 Key Laboratory of Computational Neuroscience and Brain-Inspired Intelligence, Ministry of Education, Fudan University, Shanghai, China; 4 MOE Frontiers Center for Brain Science, Fudan University, Shanghai, China; 5 School of Integrated Chinese and Western Medicine, Anhui University of Chinese Medicine, Hefei, China; 6 Institute of Integrated Chinese and Western Medicine, Anhui Academy of Chinese Medicine, Hefei, China; 7 Early Intervention Unit, Department of Psychiatry, Affiliated Nanjing Brain Hospital, Nanjing Medical University, Nanjing, China; 8 Functional Brain Imaging Institute of Nanjing Medical University, Nanjing, China; 9 Institute of Neuroscience, State Key Laboratory of Neuroscience, Chinese Academy of Sciences, Shanghai, China

**Keywords:** Molecular neuroscience, Autism spectrum disorders

## Abstract

Autism spectrum disorder (ASD) is a neurodevelopmental disorder characterized by core symptoms that consist of social deficits and repetitive behaviors. Unfortunately, no effective medication is available thus far to target the core symptoms of ASD, since the pathogenesis remains largely unknown. To investigate the pathogenesis of the core symptoms in ASD, we constructed *Shank1* P1812L-knock-in (KI) mice corresponding to a recurrent ASD-related mutation, *SHANK1* P1806L, to achieve construct validity and face validity. *Shank1* P1812L-KI heterozygous (HET) mice presented with social deficits and repetitive behaviors without the presence of confounding comorbidities. HET mice also exhibited downregulation of metabotropic glutamate receptor (mGluR1) and associated signals, along with structural abnormalities in the dendritic spines and postsynaptic densities. Combined with findings from *Shank1* R882H-KI mice, our study confirms that mGluR1-mediated signaling dysfunction is a pivotal mechanism underlying the core symptoms of ASD. Interestingly, *Shank1* P1812L-KI homozygous (HOM) mice manifested behavioral signs of impaired long-term memory rather than autistic-like core traits; thus, their phenotype was markedly different from that of *Shank1* P1812L-KI HET mice. Correspondingly, at the molecular level, *Shank1* P1812L-KI HOM displayed upregulation of AMPA receptor (GluA2)-related signals. The different patterns of protein changes in HOM and HET mice may explain the differences in behaviors. Our study emphasizes the universality of mGluR1-signaling hypofunction in the pathogenesis of the core symptoms in ASD, providing a potential target for therapeutic drugs. The precise correspondence between genotype and phenotype, as shown in HOM and HET mice, indicates the importance of reproducing disease-related genotypes in mouse models.

## Introduction

Autism spectrum disorder (ASD) is a heterogeneous neurodevelopmental disorder clinically diagnosed on the basis of social interaction and communication deficits along with restricted and repetitive patterns of behaviors, interests or activities (RRBs), as specified by the DSM-V criteria [1]. More than 70% of individuals with ASD have co-occurring medical, psychiatric, or neurological conditions [2]. The prevalence of ASD has shown a rapid upward trend, increasing from 1/150 in 2000 to 1/36 in 2020, as monitored by the Centers for Disease Control and Prevention of the United States [3]. To date, no proven medication is available to target the core symptoms of ASD [4–6]. The pathogenesis underlying the core features of ASD remains largely unknown, which needs further investigation and has important implications for therapeutic solutions.

ASD has a high degree of heritability, estimated to be as high as 90% [7, 8]. Next-generation sequencing has discovered many ASD risk genes by identifying likely gene-disrupting (LGD) mutations. Over the last decade, a series of candidate gene knockout (KO) mouse models of ASD have been established, and these models have indeed guided our understanding of the pathogenesis of ASD [9–11]. However, KO mouse models cannot mimic the pathophysiology caused by rare mutations, which play an important role in the etiology of ASD [11–14]. In particular, little attention has been paid to missense mutations because most of them are of unknown significance, even though they account for a considerable proportion of ASD cases [14, 15]. Recently, a good example was presented by Wang et al., who introduced a missense mutation of *SHANK3* into mice [16]. This mutation resulted in a small subset of phenotypes which had been previously seen in constitutive *Shank3* knockout mice and the researchers unveiled the modularity of Shank3 function in vivo of mediating a specific downstream pathway [16]. Therefore, it is necessary to adopt the strategy of knock-in (KI) mice to achieve construct validity (replicating mutations found in ASD patients) and face validity (phenotypic similarity to disease-specific symptoms) and then to explore the pathogenesis of the core symptoms of ASD using these models.

The *SHANK* family genes (*SHANK1/2/3*) are well-known ASD-related genes, encoding multidomain proteins that function as master regulators at the postsynaptic density (PSD) in glutamatergic synapses [17, 18]. A large body of evidence from human and animal model studies demonstrates that glutamatergic synaptic dysfunction represents a common pathology underlying ASD [10, 19–21]. In our previous work, we identified an ASD-specific recurrent missense mutation of *SHANK1*, c.2621 G > A (p.R874H), and we generated a corresponding KI mouse model (*Shank1* R882H-KI mice) to illuminate the underlying neurological and molecular mechanisms [22]. The R882H-KI mice manifested two autistic-like core symptoms, namely, social disability and repetitive behaviors, without the presence of confounding comorbidities. Importantly, significant downregulation of mGluR1-IP3R1-calcium signaling in specific brain regions of KI mice was recognized to be exclusively related to the core symptoms. That study was the first to identify the vital role of mGluR-IP3R-calcium signaling in the core symptoms of ASD. On that basis, a question naturally arose as to whether the change in mGluR1-mediated signaling exists specifically for the mutation of R874H or universally for other mutations in *SHANK1*. In our previous work, we also found another recurrent *SHANK1* mutation, c.5417 C > T (p.P1806L), in two unrelated patients with ASD [22]; this mutation is located in the proline-rich (PRO) region. In vitro morphological analyses of P1806L showed severely impaired maturation of dendritic spines. Combining the evidence from human genetics and in vitro functional assessments, we considered P1806L to be worthy of more detailed investigation in a mouse model. Such a model would provide an excellent opportunity to explore potential commonalities and differences in the molecular underpinnings of autistic symptoms between two different *SHANK1* missense mutations.

Accordingly, in this study, we developed a KI mouse model carrying the P1812L mutation, which corresponds to the P1806L mutation in ASD patients. We comprehensively characterized the behavioral outcomes, molecular processes and synaptic phenotypes of *Shank1* P1812L-KI mice. Combining these findings with those from *Shank1* R882H-KI mice, we further identified mGluR1-mediated signaling dysfunction as the pivotal molecular mechanism underlying the core symptoms of ASD.

## Materials and methods

### Construction of *Shank1* P1812L-KI mice

*Shank1* P1812L-KI mice were generated by CRISPR/Cas9 as previously described [23]. Mice were kept under a 12 h/12 h light/dark cycle and provided with food and water ad libitum. Heterozygous mice were bred to obtain littermates of three genotypes, male mice from which were used for all experiments. Offspring were genotyped on postnatal day 14 (P14) and weaned on P21. The genotypes of the mice were established by Sanger sequencing of PCR products from tail biopsy DNA. The sequences for CRISPR/Cas9 editing and genotyping are listed in Supplementary Table 1. This study was approved by the Ethics Committee of Animal Experimentation at Fudan University. Procedures were carried out according to the guidelines of the Care and Use of Laboratory Animals proposed by Fudan University and Shanghai Municipality, P.R. China (Permit Number: SYXK (hu) 2020-0032).

### Behavioral tests

Behavioral tests were performed with age-matched littermates (>8 weeks) during 10:00‒16:00 using previously described protocols [24–32]. The mice were gently handled for 5 min/day for 7 consecutive days prior to testing. All mice were allowed to adapt to the testing room for 60 min before the start of each behavioral test.

#### Three-chamber social interaction and social novelty test

A rectangular box (61.5 × 60.5 × 31 cm^3^) was used; this apparatus was made up of a 20.5 cm-wide center chamber and two 20.5 cm-wide side chambers. Doors within two partitions allowed access to all chambers. Age-matched male strangers unfamiliar to the subjects were habituated to the apparatus for 30 min/day for 3 consecutive days. In the adaptation phase, the subject mice explored for 10 min with the doors closed, followed by another 10 min with the doors open. In the interaction phase, stranger mouse #1 (S#1) was placed in an inverted wire cup in one side chamber, and an empty inverted wire cup (novel object, O) was placed in the other side. The subject mouse was allowed to explore for 10 min. In the novelty preference phase, stranger #2 (S#2) was placed in a cup in the other side chamber. The subject was placed in the apparatus again for 10 min. The positions of S#1 and O or S#2 were changed between trials. The parameters were analyzed by EthoVision XT (Noldus, USA), including the total number of entries into the side chambers, the time spent in each side chamber and the time spent sniffing each target. Data were excluded if the subject mouse had not visited either side chamber in any phase.

#### Marble-burying (MB) test

Polycarbonate rat cages (47 × 26 × 31.5 cm^3^) were filled with corncob bedding to a depth of 5 cm, and then 20 glass marbles (1.5 cm) were placed on the surface in 4 × 5. The subject mouse was placed in a corner of the cage and allowed to explore for 30 min with the cage lid in place. At the end of the test, the number of marbles with over 1/2 volume buried was scored for each mouse.

#### Open field (OF) test

Each subject was placed in the center of an apparatus (40 × 40 × 40 cm^3^) for an exploration period of 30 min. The movement time, total distance traveled and distance traveled in the center (1/4 of the total area) were recorded and analyzed with EthoVision XT (Noldus, USA). The center distance ratio was calculated as the distance the subject traveled in the center divided by the total distance traveled.

#### Light-dark box (LDB) test

The apparatus (44 × 17.5 × 26 cm^3^) was separated equally into a light compartment (transparent walls, 400 lux) and a dark compartment (opaque black walls, 4 lux). An electrically operated door (10 cm × 5 cm) at the bottom of the partition allowed access to two compartments, and an infrared detector was set to detect crossings. The subject mouse was placed in the light compartment away from the partition and allowed to explore for 10 min. The number of transitions between the two compartments and the time spent in the light compartment were collected and analyzed with MED-SYST-VFC-USB (Med Associates, USA).

#### Elevated plus maze (EPM) test

The apparatus comprised two open arms (30 × 7 × 0.5 cm^3^) and two closed arms (30 × 7 × 15.5 cm^3^) extending from a center (7 × 7 × 0.5 cm^3^). It was elevated 50 cm above the floor. The subject mouse was placed in the center facing a closed arm and allowed to explore for 10 min. Sessions were analyzed with EthoVision XT (Noldus, USA) to obtain the number of total entries into both arms, the time spent in the open arms and the number of entries into the open arms.

#### Barnes maze (BM) test

The apparatus for the test was a circular platform (122 cm in diameter) elevated 80 cm above the floor and containing 40 holes (each 5 cm in diameter); the surface was illuminated at 250 lux as a stimulus, and geometric shapes were placed on the walls as visual cues. A box was placed under a designated target escape hole. The subject mouse was habituated to the maze and then performed 15 trials in 4 consecutive days as previously described [22]. Probe trials of 90 s for assessing short-term and long-term retention were performed on Day 5 and Day 12, respectively. All sessions were analyzed by EthoVision XT (Noldus, USA). The parameters measured included the number of total errors and the latency to enter the escape box during the training period, as well as the number of total errors and the percentage of time spent in the target quadrant during the probe trial.

### Golgi staining

Histological analysis of dendritic spines was performed with an FD Rapid GolgiStain Kit (FD Neurotechnologies, USA) according to a modified protocol, as previously described [22]. Mice (5–6 weeks) were anesthetized, and the brains were dissected to obtain tissue blocks containing the hippocampi. Morphometric reconstruction and calculations for the collected neurons were performed using ImageJ (NIH, USA) and Neurolucida (MBF Bioscience, USA). Dendritic spine density, spine length and spine head width were measured 10–15 μm away from the beginning of the secondary apical dendrites of pyramidal neurons. The spine density was defined as the average number of spines per 10 microns of dendritic length.

### Transmission electron microscopy (TEM)

TEM was carried out as previously described [22]. Mice (5–6 weeks) were anesthetized, and hippocampal tissues were dissected and fixed. Coronal sections (50 µm) were cut on a vibratome (VT1200S, Leica, Germany) and processed for embedment. Ultrathin (70 nm) sections were cut on an ultramicrotome (EM UC7, Leica, Germany) and poststained with uranyl acetate and Sato’s lead citrate. Electron micrographs were randomly acquired from CA1 at ×15,000 magnification with an electron microscope (HT7800, Hitachi, Japan) at an accelerating voltage of 120 kV. Asymmetric synapses with clear structures were assessed for PSDs [33–35]. Measurements were performed with ImageJ. The length was measured directly, while the average thickness was calculated as the area divided by the length. For perforated PSDs, the entire length of all fragments was summed together.

### Subcellular fractionation

Subcellular fractions were extracted at 4 °C from the mouse (5–6 weeks) cerebral cortex tissues using previously described protocols with some modifications [36, 37]. Protease and phosphatase inhibitors (APExBIO, USA) were used at each step. Tissue was homogenized in HEPES-buffered sucrose (0.32 M sucrose, 4 mM HEPES, pH 7.4) and centrifuged at 1000 × *g*. The supernatant was centrifuged at 12,000 × *g* to obtain a crude synaptosomal pellet.

### Immunoblot analysis and antibodies

Subcellular fractions were lysed in a 4:1 mixture of 0.54% Triton X-100/0.5% SDS in 50 mM HEPES/2 mM EDTA solution containing protease and phosphatase inhibitors. Lysates were quantified with a BCA assay. Western blotting was performed on PVDF membranes with an ECL kit (Tanon, China) using standard protocols. Multiple primary antibodies purchased from suppliers are listed in Supplementary Table 2. Quantitative analysis of the grayscale value of each band was carried out with ImageJ, and the relative levels of specific proteins were normalized to that of β3-Tubulin.

### Statistics

Statistical analyses and graphical illustrations were performed using GraphPad Prism or R. Student’s *t* test, one-way ANOVA, the Kruskal‒Wallis test, or repeated-measures ANOVA was used as indicated in the figure legends. Tukey’s or Dunnett’s post hoc tests following ANOVA and the Kolmogorov‒Smirnov test following the Kruskal‒Wallis test were applied when appropriate. The data are presented as the mean ± SE. All statistical assessments were two-sided and used a significance threshold of 0.05.

## Results

### Generation of *Shank1* P1812L-KI mice

To reveal the in vivo functional significance of the ASD-associated *SHANK1* P1806L mutation, we created *Shank1* P1812L-KI mice using a CRISPR/Cas9 strategy without off-target mutations. Both heterozygous (HET) and homozygous (HOM) mice were viable, fertile, free of any obvious developmental defects, and similar in body weight to wild-type (WT) mice (Supplementary Fig. 1A). No differences of the expressions of Shank1/2/3 proteins in the brain regions of cortex and hippocampus were found in mutant mice (Supplementary Fig. 2).

### Only *Shank1* P1812L-KI HET mice, not HOM mice, exhibit autistic-like core behavioral symptoms

**Fig. 1:**
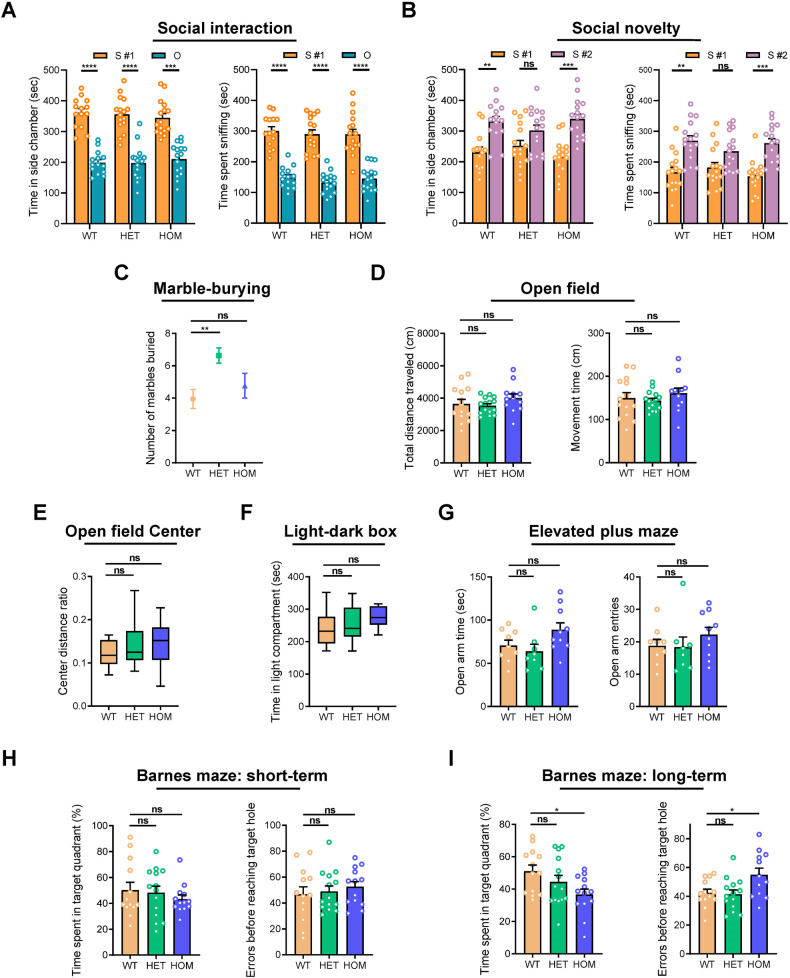
Autistic-like core behaviors in *Shank1* P1812L-KI HET mice but not in HOM mice. Social novelty deficits in P1812L-KI HET mice in the three-chamber social test (*n* = 14–16 for each genotype) (**A**, **B**). **A** Social interaction. S #1, a stranger mouse. O, a novel object. **B** Social novelty preference. S #1, a familiar, previously investigated mouse. S #2, a novel unfamiliar mouse. **C** Increased RRBs in P1812L-KI HET mice in the marble-burying test (*n* = 17–20 for each genotype). **D** Normal locomotion in P1812L-KI mice in the open field test (*n* = 12–15 for each genotype). No anxiety-like behavior in P1812L-KI mice, as measured by the center activity in the open field test (**E**), the light-dark box test (**F**) (*n* = 12–14 for each genotype) and the elevated plus maze test (**G**) (*n* = 8–10 for each genotype). **H** Normal short-term (Day 5) spatial memory in P1812L-KI mice in the Barnes maze (*n* = 13–14 for each genotype). **I** Deficits in long-term (Day 12) spatial memory in P1812L-KI HOM mice, as indicated by both parameters. Paired Student’s *t* test for (**A**, **B**). One-way ANOVA for (**C**–**I**). All data are presented as the mean ± SE. ns, no significance, *P < 0.05, **P < 0.01, ***P < 0.001, ****P < 0.0001.

Two core behavioral features of ASD were evaluated in *Shank1* P1812L-KI mice. Social behaviors were examined using the three-chamber test. During the social interaction session, mice of all genotypes performed normally, preferring to spend more time with the strange mouse than the novel object (Fig. 1A). In the session for social novelty, P1812L-KI HET mice showed no difference in the amount of time spent for either the unfamiliar mouse (stranger #2) or the familiar mouse (stranger #1), as reflected by the similar amounts of time spent in the two side chambers and similar amounts of time spent sniffing the two mice (Fig. 1B), suggesting impaired social novelty preference [31]. Unexpectedly, P1812L-KI HOM mice did not exhibit this defect. Total entries into the two side chambers, a within-task control for the general level of exploratory behavior, did not differ among the three genotypes (Supplementary Fig. 1B and C). In the marble-burying test for measuring RRBs, P1812L-KI HET mice buried significantly more marbles than WT littermates did (Fig. 1C), indicating increased RRBs. Unexpectedly, the performance of P1812L-KI HOM mice was comparable to that of WT controls.

To investigate motor and anxiety-like behaviors, we conducted the OF, LDB and EPM tests. Both total distance and movement time in the OF showed that locomotion was not affected in mutant mice (Fig. 1D). This finding was supported by other measures, including total entries in the three-chamber test (Supplementary Fig. 1B and C), total transitions between compartments in the LDB (Supplementary Fig. 1D), and total entries into arms in the EPM (Supplementary Fig. 1E). No anxiety-like behavior was observed in mutant mice, as shown by the absence of any differences in the center distance ratio in the OF (Fig. 1E), the time spent in the light compartment in the LDB (Fig. 1F) or the open arm time/entries in the EPM (Fig. 1G) among genotypes.

Cognitive performance was assessed through the BM task. Mice of three genotypes were able to learn the task at the end (Supplementary Fig. 1F), showing normal acquisition performance. During probe trials, P1812L-KI HOM mice performed poorly in locating the target quadrant on Day 12 but not Day 5 compared with mice of other genotypes, as shown by both quadrant time and errors (Fig. 1H, I), indicating long-term memory impairment.

Collectively, these results suggest that *Shank1* P1812L-KI HET mice but not HOM mice present with autistic-like core behavioral symptoms of social deficits and repetitive behaviors without comorbidities. Contrary to expectations, *Shank1* P1812L-KI HOM mice merely display impaired long-term spatial memory.

### Morphological abnormalities of glutamatergic synapses in mutant mice, especially *Shank1* P1812L-KI HET mice

**Fig. 2:**
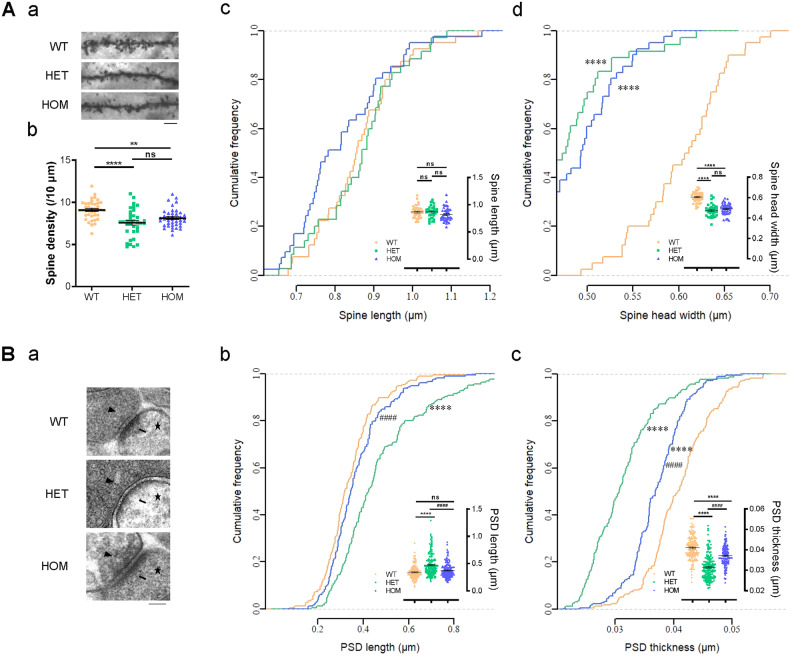
Morphological abnormalities in glutamatergic synapses. **A** Decreased density and abnormal morphology of CA1 dendritic spines in *Shank1* P1812L-KI mice as determined by Golgi staining (35-41 neurons and >1400 spines from three mice per genotype). **A**, a Representative images of secondary dendrites. The scale bar is 5 µm. **A**, b Reduced spine density in P1812L-KI mice. **A**, c and d Reductions in spine head width but not spine length in P1812L-KI mice. **B** Ultrastructure of CA1 glutamatergic synapses observed by TEM ( > 160 PSDs from three mice for each genotype). **B**, a Representative TEM images. Arrowheads, synaptic vesicles; arrows, PSDs; stars, dendritic spines. The scale bar represents 100 nm. **B**, b and c Increased length and reduced thickness of PSDs, especially in P1812L-KI HET mice. One-way ANOVA for scatter plots. Kruskal‒Wallis test for cumulative frequency plots. All data are presented as the mean ± SE. ns, no significance. **P < 0.01, ****P < 0.0001 for groups compared with WT. ^####^*P* < 0.0001 for groups compared with HET.

To test how the P1806L mutation affects glutamatergic synapse morphology, we first examined CA1 pyramidal neurons using Golgi staining and found spine maturation defects in mutant mice (Fig. 2A, a). Spine density was significantly reduced in P1812L-KI HET mice (−16.9%) and HOM mice (−10.8%) compared with WT littermates (9.10 ± 0.17 in WT, 7.56 ± 0.28 in HET and 8.12 ± 0.17 in HOM per 10 μm; Fig. 2A, b). A pronounced reduction in spine head width was found in P1812L-KI HET mice (−21.7%) and HOM mice (−18.3%) compared with WT controls owing to the increased percentage of small dendritic spines (0.60 ± 0.01 μm in WT, 0.47 ± 0.01 μm in HET and 0.49 ± 0.01 μm in HOM; Fig. 2A, d). No difference was found in spine length among mice of the three genotypes (0.86 ± 0.02 μm in WT, 0.86 ± 0.02 μm in HET and 0.82 ± 0.02 μm in HOM; Fig. 2A, c).

Furthermore, the ultrastructure of glutamatergic synapses was analyzed by TEM (Fig. 2B, a). Notably, both the length (+38.6%) and thickness (−23.3%) of PSDs were dramatically altered in P1812L-KI HET mice (0.47 ± 0.02 μm for length, 0.031 ± 0.0005 μm for thickness; Fig. 2B, b, c) compared with WT mice (0.34 ± 0.01 μm for length, 0.041 ± 0.0004 μm for thickness). A change in thickness (−9.4%) but not length was observed in P1812L-KI HOM mice (0.37 ± 0.01 μm for length, 0.037 ± 0.0004 μm for thickness); this change was smaller than that observed in HET mice.

Taken together, these data reflect morphological abnormalities in synapses and indicate that these abnormalities are more severe in P1812L-KI HET mice than in HOM mice.

### Downregulated mGluR1-mediated signaling underlying autistic-like core symptoms

**Fig. 3:**
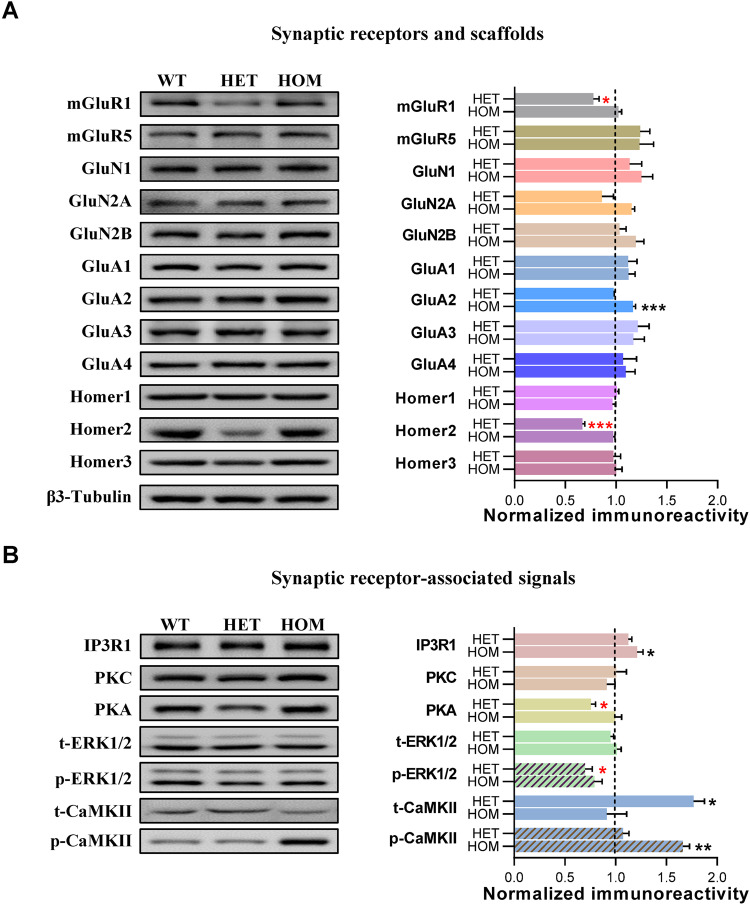
Downregulation of mGluR and downstream signaling pathways in glutamatergic synapses in *Shank1* P1812L-KI HET mice but not in HOM mice. **A** mGluR1 and Homer2 were decreased in P1812L-KI HET mice. GluA2 was increased in P1812L-KI HOM mice. **B** PKA and p-ERK1/2 were decreased in P1812L-KI HET mice. IP3R1 and p-CaMKII were increased in P1812L-KI HOM mice. For (**A**) and (**B**), the left side shows representative bands for the indicated proteins; each lane was loaded with protein samples from an individual mouse. The right side shows the corresponding statistical results, normalized to WT levels. β3-Tubulin, serving as a loading control, is displayed in (**A**). The results were obtained from at least three mice per genotype and performed at least in duplicate. All data are presented as the mean ± SE. *P < 0.05, **P < 0.01, ***P < 0.001 for groups compared with WT, by one-way ANOVA.

To investigate alterations in the PSD molecular network related to behavioral phenotypes, the crude synaptosomal fraction from the cortex, where *Shank1* is highly enriched [38, 39], was isolated for semiquantitative immunoblotting. Changes in glutamate receptors differed between P1812L-KI HET mice and HOM mice in that a significant decrease in mGluR1 was observed in P1812L-KI HET mice, while an increase in the AMPA receptor (AMPAR) subunit GluA2 was found in P1812L-KI HOM mice (Fig. 3A). Furthermore, the abundance of Homer2, a scaffold linking Shank1 and mGluRs, was found to be reduced in HET mice but normal in HOM mice (Fig. 3A).

Next, glutamate-receptor-mediated downstream signals were examined. Key molecules associated with mGluR1 were found to be significantly decreased, including PKA and p-ERK1/2, in P1812L-KI HET mice (Fig. 3B). Interestingly, the level of active CaMKII (p-CaMKII) was not influenced despite a dramatic increase in the total amount of t-CaMKII in P1812L-KI HET mice (Fig. 3B). Unexpectedly, we found another alteration profile in P1812L-KI HOM mice, in which IP3R1 and p-CaMKII were significantly elevated while other proteins showed no change (Fig. 3B).

These results suggest that the downregulation of mGluR1-associated signaling may contribute to the core behavioral features of P1812L-KI HET mice, while a completely distinct alteration of PSD proteins, including an increase in GluA2, may underlie the impaired memory observed in P1812L-KI HOM mice.

## Discussion

In this study, we in vivo assessed and confirmed the pathogenicity of a recurrent *SHANK1* missense mutation, c.5417 C > T (p.P1806L), identified in patients with ASD. *Shank1* P1812L-KI HET mice presented with autistic-like core symptoms consisting of social deficits and repetitive behaviors, which were not accompanied by comorbid abnormalities. The levels of mGluR1 and the associated downstream molecules PKA and p-ERK1/2 were significantly decreased in P1812L-KI HET mice. Our investigation links the downregulation of mGluR1-mediated signaling specifically to the core symptoms of ASD in a mouse model with construct validity and face validity.

**Fig. 4:**
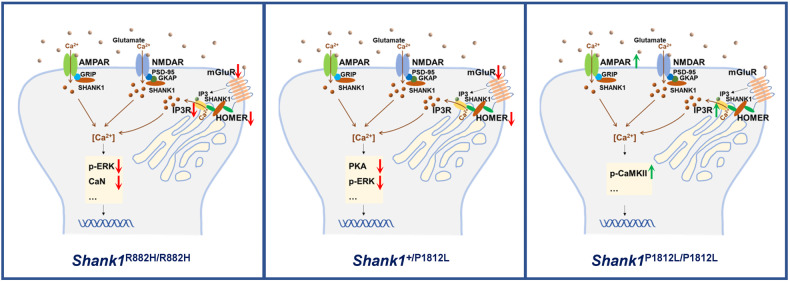
Schematic diagram illustrating the specific signaling dysregulated in *Shank1* mutant mice. *Shank1* R882H-KI HOM mice (R882H/R882H), *Shank1* P1812L-KI HET mice (+/P1812L) and HOM mice (P1812L/P1812L). The downregulated proteins are shown with a downward arrow in red; the upregulated proteins are shown with an upward arrow in green. For ERK and CaMKII, the phosphorylated forms of the proteins were considered.

Behavioral characterization of *Shank1* P1812L-KI mice showed that HET mice manifested two core traits of ASD without comorbidities such as abnormal locomotion or anxiety-like behaviors, which are confounding factors for the interpretation of outcomes in assays for social behaviors and RRBs [25, 27, 40, 41]. Two independent lines, *Shank1* P1812L-KI HET mice and *Shank1* R882H-KI mice, exhibit the same phenotypes, suggesting that these phenotypes truly reflect the pathogenic effect induced by *SHANK1* mutations and providing an excellent resource to unveil the mechanisms of the core features of ASD. In this study, not only mGluR1 but also its downstream signaling molecules PKA and p-ERK1/2 [42] were robustly downregulated, specifically corresponding to social deficits and RRBs in P1812L-KI HET mice. Interestingly, these results coincide with the findings of decreased mGluR1 and its associated molecules, including p-ERK1/2 and Ppp3ca/Ppp3cb, in R882H-KI mice (Fig. 4) [22]. In addition, the reduction in Homer2 may be responsible for the change in mGluR1 because Homers directly tether mGluRs to Shanks through the PRO domain [43–45]. As found in our previous work, Homer2 is highly expressed in the cortex [22], and a specific reduction in Homer2, with no decrease in Homer1 or Homer3, reflects the subtype-specific expression profile of Homer proteins in brain regions. Therefore, these findings from two mouse lines illustrate the perfect correlation between phenotypes and molecular underpinnings, identifying the downregulation of mGluR1-mediated signaling as the mechanism underlying the core symptoms of ASD (Fig. 4).

Intriguingly, a completely distinct behavioral profile was observed in *Shank1* P1812L-KI HOM mice, in which long-term memory was severely hampered while autistic-like core symptoms were absent. The dissection of the PSD network showed alterations in the ionotropic receptor subunit GluA2 but not in metabotropic receptors. Therefore, P1812L-KI HOM mice mainly showed increased GluA2 levels and impaired long-term memory performance. The upregulation of the GluA2 subunit and IP3R1 may contribute to changes in the intracellular calcium (Ca^2+^) concentration because GluA2 renders AMPARs impermeable to Ca^2+^ [46–49], while IP3R1 is responsible for Ca^2+^ release from the endoplasmic reticulum (ER) [50]. On the other hand, both of them may be linked to long-term potentiation (LTP)/long-term depression (LTD) deficits, since GluA2 and IP3R1 knockout mice exhibit altered LTP/LTD [51, 52]. CaMKII is a critical mediator that links Ca^2+^ signals to persistent changes in neuronal physiology [53–57]. p-CaMKII elevation represents a contribution to synaptic plasticity. These factors could have a profound impact on memory, but the exact mechanism remains to be further explored. The nonoverlapping behavioral symptoms and the differential severity of synaptic anomalies between P1812L-KI HOM mice and P1812-KI HET mice could be explained by this pattern of altered proteins, which has nothing in common with the pattern in HET mice. A similar phenomenon was also found in the *Nlgn1* P89L-KI mouse model of ASD, in which homozygotes displayed milder phenotypes, i.e., typical sociability, than heterozygous mice [58]. However, this puzzling issue was not resolved in that study. Here, our work provides a possible explanation that diverse patterns of molecular mechanisms underlie the differential behavioral and synaptic features of the two genotypes.

To date, numerous lines of *Shank* family gene knockout mice have been established and characterized [27, 33, 37, 39, 59–71]. Despite the fact that most of these mouse models do not carry human mutations, they provide important insights into the pathophysiology of ASD. The presence of autistic-like core and/or ancillary behavioral deficits is always linked to the existence of glutamate receptor dysregulation and synaptic dysfunction [37, 39, 60–62, 66–69]. However, the specific relationships between them remain difficult to determine. No changes of receptors have been detected in brains in *Shank1*-KO mice [33]. The receptors commonly found to have prominent changes in *Shank2*-KO or *Shank3*-KO mice were NMDAR subunits and/or AMPAR subunits [39, 60–62, 66, 67]. In contrast, the contribution of mGluRs to ASD-related phenotypes in these mice was not fully investigated. Here, in our work on KI mice mimicking patient-derived P1806L as well as R874H, we successfully extracted the core domains from complex ASD traits and identified a dominant mGluR-mediated pathway responsible for the core subset. Our work emphasized the pathogenic effect of mGluR-mediated signaling on the core symptoms in ASD-linked *SHANK1* mutations, compensating for the limitation that *Shank1*-KO mice did not effectively identify the core phenotypes [27, 33, 59].

There are several limitations in this study. Investigation of rescue strategies would provide more details to understand the pathological mechanism underlying ASD core features, which is not included in this work. Furthermore, the neural circuitry of candidate brain regions underlying core behaviors in *Shank1* P1812L-KI mice is totally unknown, which needs to be explored in the future. Another limitation is that our strategy for choosing cortex for biochemical analysis and hippocampus for spine morphological analysis is favorable to acquire more results in brain regions efficiently, and however, analyzing all the regions will be a perfect situation.

In summary, the present study effectively identified the pathogenicity of the *SHANK1* P1806L mutation in ASD. These results are consistent with previous findings in *Shank1* R882H-KI mice [22], providing further evidence for the causative role of *SHANK1* mutations in ASD via the downregulation of mGluR1-mediated signaling, especially with respect to the core phenotypes. The validation of mGluR1-signaling hypofunction as the cause of the core symptoms of ASD will provide a prospective target for the therapy.

### Supplementary information

Supplementary file
