# Supplementary material for: Downregulation of mGluR1-mediated signaling underlying autistic-like core symptoms in Shank1 P1812L-knock-in mice
Source: Transl Psychiatry. 2023 Oct 25;13:329. doi: 10.1038/s41398-023-02626-9 (PMC10600164; doi:10.1038/s41398-023-02626-9)
Supplement: Supplementary file 1 — Supplementary file [file 41398_2023_2626_MOESM1_ESM.docx]

**Supplementary Information**

**Downregulation of mGluR1-mediated signaling underlying autistic-like core symptoms in *Shank1* P1812L-knock-in mice**

Yue Qin; Xiao-Yong Zhang; Yanyan Liu; Zehan Ma; Shuo Tao; Ying Li; Rui Peng; Fei Wang; Jiucun Wang; Jianfeng Feng; Zilong Qiu; Li Jin; Hongyan Wang; Xiaohong Gong

**Summary**

The supplementary information file includes sections of Supplementary Tables and Supplementary Figures.

**Supplementary Tables**

**Supplementary Table 1. DNA sequences for construction of *Shank1* P1812L-knock-in mice**

| **Sequence Name** | **Forward primer/Reverse primer or sequence (5’-3’)** | **Purpose** |
| --- | --- | --- |
| sgRNA | TGTTCTGGACCCTCAACGGC | CRISPR/Cas9 editing |
| ssODN | CGTCTCTGTGACAGGAGCTGGAACAGATGGGCTACTGGCCTTGAGTGCTTGTTCTGGAttgTCAACGGCAGGTGTGGCAGGGGGTCCCGTGGCGGTTGAGCCAGAAGTCCCGCCTG | CRISPR/Cas9 editing |
| GT-1812 | CTGGCCTTGAGTGCTTGTTC / TGTAACTCCGAGGCTCTATGG | Genotyping in mice |

**Supplementary Table 2. Information for primary antibodies used in immunoblotting**

| **Antibody** | **Type** | **Provider** | **Cat.No** | **Dilution** |
| --- | --- | --- | --- | --- |
| β3-Tubulin | Mouse monoclonal | Abcam, UK | ab78078 | 1:10000 |
| mGluR1 | Rabbit monoclonal | Cell Signaling, USA | 12551 | 1:1000 |
| mGluR5 | Rabbit monoclonal | Beyotime, China | AF1744 | 1:1000 |
| GluA1 | Rabbit monoclonal | Beyotime, China | AF2473 | 1:1000 |
| GluA2 | Rabbit polyclonal | Proteintech, USA | 11994-1-AP | 1:2000 |
| GluA3 | Rabbit polyclonal | ABclonal, China | A1195 | 1:500 |
| GluA4 | Rabbit polyclonal | Proteintech, USA | 23350-1-AP | 1:500 |
| GluN1 | Rabbit monoclonal | Abcam, UK | AB109182 | 1:4000 |
| GluN2A | Rabbit polyclonal | Proteintech, USA | 19953-1-AP | 1:1000 |
| GluN2B | Rabbit polyclonal | Proteintech, USA | 21920-1-AP | 1:2000 |
| Homer1 | Rabbit polyclonal | Proteintech, USA | 12433-1-AP | 1:1000 |
| Homer2 | Rabbit polyclonal | Proteintech, USA | 11143-1-AP | 1:1000 |
| Homer3 | Rabbit polyclonal | Proteintech, USA | 16624-1-AP | 1:1000 |
| t-CaMKII | Rabbit polyclonal | Proteintech, USA | 20666-1-AP | 1:1000 |
| p-CaMKII | Rabbit polyclonal | Affinity, USA | AF3493 | 1:1000 |
| PKC | Rabbit polyclonal | Proteintech, USA | 12919-1-AP | 1:2000 |
| t-ERK1/2 | Rabbit monoclonal | Cell Signaling, USA | 4695 | 1:4000 |
| p-ERK1/2 | Rabbit monoclonal | Cell Signaling, USA | 4370 | 1:2000 |
| PKA | Rabbit polyclonal | Cell Signaling, USA | 4782 | 1:1000 |
| Shank1 | Rabbit polyclonal | NOVUS | NB300-167 | 1:500 |
| Shank2 | Rabbit polyclonal | Abmart | PA2556 | 1:1000 |
| Shank3 | Rabbit polyclonal | Abmart | TD9901 | 1:1000 |

**Supplementary Figures**


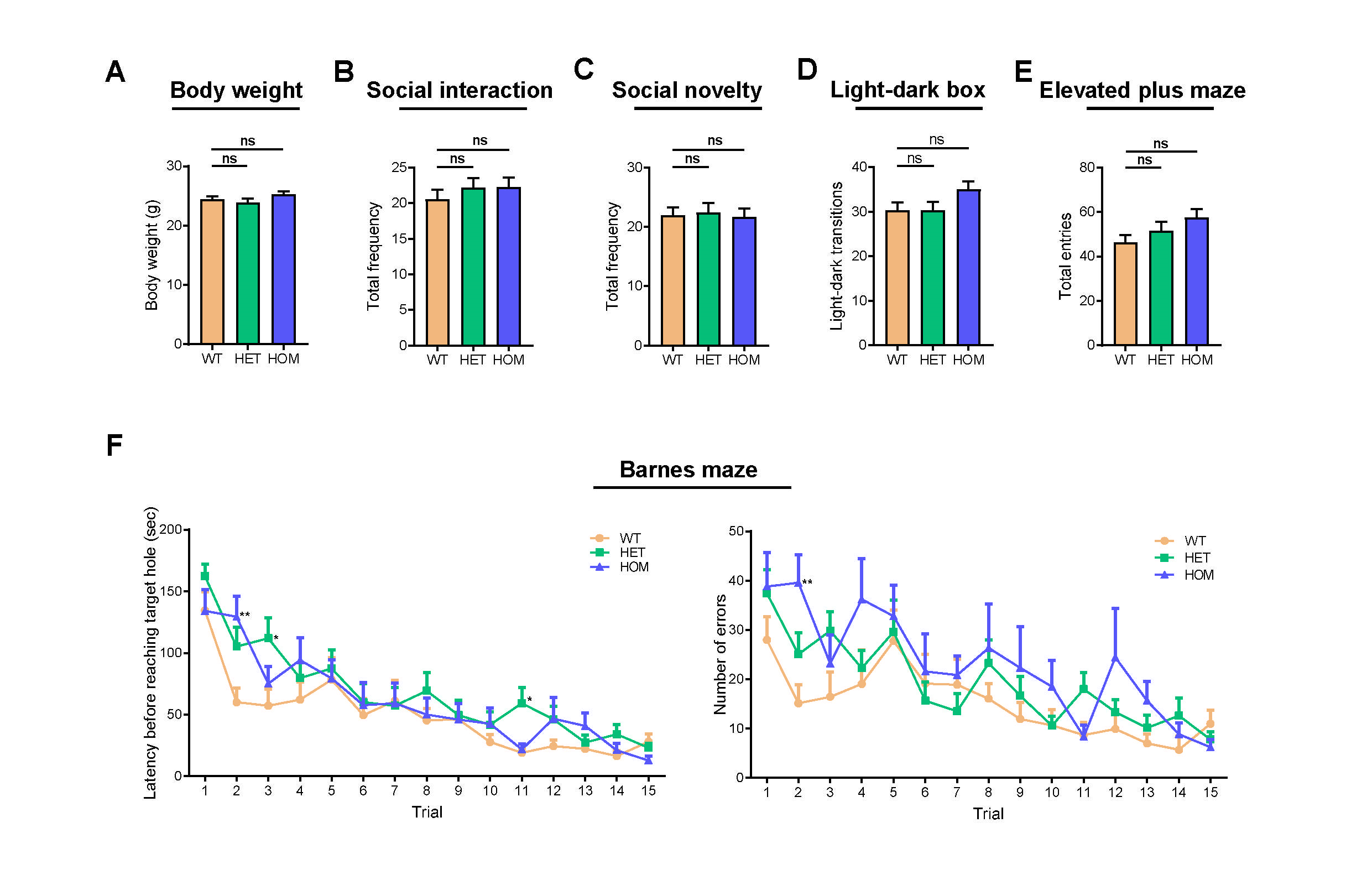


**Supplementary Figure 1** (A) No difference in body weights of animals of three genotypes at 12-15 weeks (n=12-16 for each genotype). (B and C) No differences in numbers of entries into side chambers in the three-chamber social tests (n=14-16 for each genotype). (D) No difference in total transitions between two compartments in the light-dark box test (n=12-14 for each genotype). (E) No differences in total entries in the elevated plus maze test (n=8-10 for each genotype). (F) Normal performance of spatial learning acquisition, except in trial 2, in the Barnes maze (n=13-14 for each genotype), as measured both by the latency before reaching the target hole (genotype, *P*=0.0479, trial, *P*<0.0001, interaction, *P*=0.2178) and the number of errors (genotype, *P*=0.0127, trial, *P*<0.0001, interaction, *P*=0.4601). One-way ANOVA for (A)-(E). Repeated measures ANOVA for (F). All data are presented as the mean ± SE. ns, no significance, ^*^*P*<0.05, ^**^*P*<0.01.


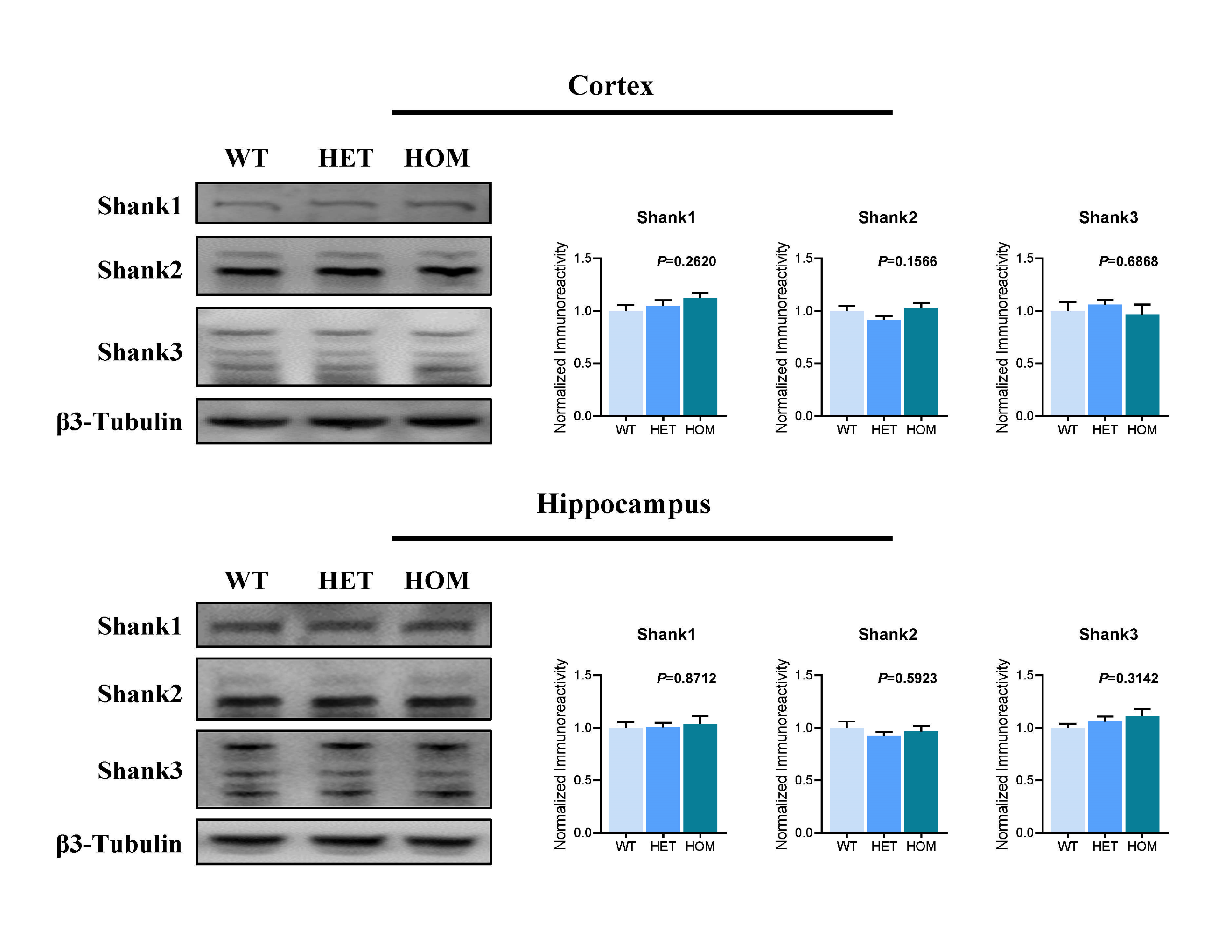


**Supplementary Figure 2** No differences of Shank1/2/3 were found in the cortex or hippocampus in mutant mice. The left side shows representative bands for the indicated proteins; each lane was loaded with protein samples from an individual mouse. The right side shows the corresponding statistical results, normalized to WT levels, with β3-Tubulin serving as a loading control. The results were obtained from at least three mice per genotype. All data are presented as the mean ± SE and compared by one-way ANOVA.
